# Case Report: Oral Cimetidine Administration Causes Drug-Induced Immune Hemolytic Anemia by Eliciting the Production of Cimetidine-Dependent Antibodies and Drug-Independent Non-specific Antibodies

**DOI:** 10.3389/fmed.2021.723167

**Published:** 2021-09-24

**Authors:** Yuanjun Wu, Yong Wu, Yanli Ji, Yanhui Liu, Dongsheng Wu, Jiajie Liang, Ganping Guo, Baochan Chen

**Affiliations:** ^1^Department of Blood Transfusion, Dongguan Maternal and Child Health Hospital, Dongguan, China; ^2^Department of Blood Transfusion, Dongguan Tungwah Hospital, Dongguan, China; ^3^Institute of Clinical Blood Transfusion, Guangzhou Blood Center, Guangzhou, China; ^4^Dongguan Institute of Reproductive and Genetic Research, Dongguan Maternal and Child Health Hospital, Dongguan, China; ^5^Department of Hematology, Dongguan Tungwah Hospital, Dongguan, China

**Keywords:** cimetidine, drug-induced antibodies, drug dependent antibodies, drug-independent antibodies, drug-induced immune hemolytic anemia (DIIHA)

## Abstract

Previously, it was reported that multiple patients had hemolytic anemia associated with cimetidine administration, while only one patient who had received intravenous cimetidine was serologically diagnosed with drug-induced immune hemolytic anemia (DIIHA) caused by cimetidine-dependent antibodies. However, the ability of oral cimetidine intake to induce the production of antibodies has not been examined. In this study, we report a 44-year-old male patient in whom oral cimetidine administration resulted in cimetidine-dependent antibodies and drug-independent non-specific antibodies, leading to the development of DIIHA. Serological tests showed that the results of direct antiglobulin test (DAT) for anti-IgG (3+) and anti-C3d (1+) were positive. The IgM and IgG cimetidine-dependent antibodies (the highest total titer reached 4,096) were detected in the plasma incubated with O-type RBCs and 1 mg/mL cimetidine or the plasma incubated with cimetidine-coated RBCs. IgG-type drug-independent non-specific antibodies were detected in blood samples collected at days 13, 34, 41, and 82 post-drug intake. This is the first study to report that oral administration of cimetidine can elicit the production of cimetidine-dependent antibodies, leading to DIIHA, and the production of drug-independent non-specific antibodies, resulting in hemolytic anemia independent of cimetidine. Presence of pathogenic antibodies were detectable longer than 41 days. This suggests that patients with DIIHA caused by cimetidine need to be given necessary medical monitoring within 41 days after cimetidine intake.

## Introduction

More than 140 drugs are associated with drug-induced immune hemolytic anemia (DIIHA) (1–9). The pathogenic mechanism of DIIHA involves the damage of red blood cells (RBCs) by drug-induced (drug-dependent and –independent) antibodies or non-immune protein adsorption (NIPA) (10–13). NIPA, which can be detected based on the positive direct antiglobulin test (DAT) result, leads to slow, mild, or almost undetectable hemolysis (13–15). Most cases of DIIHA are reported to be caused by drug-induced antibodies (12). DIIHA is diagnosed based on the manifestation of hemolysis after drug treatment and the detection of target drug-induced antibodies (12).

Cimetidine binds competitively with histamine H_2_ receptors on parietal cells in the gastric wall and consequently inhibits gastric acid secretion. Hence, cimetidine has been widely used for treating gastric and duodenal ulcers and reflux esophagitis, as well as for preventing stress ulcers. Based on the immunomodulatory effect of cimetidine, it has been used in the treatment of warts, ulceration, and mastocytosis in dermatology (16, 17). Cimetidine has been shown to inhibit heme biosynthesis and results in symptomatic improvement in patients with acute intermittent porphyria and porphyria cutanea tarda. In recent years, cimetidine has been used as a new method of erythropoietic protoporphyria treatment (18). Cimetidine tablets and capsules have been used commonly as non-prescription drugs without medical supervision (19).

Since 1979, several cases of immune hemolytic anemia (IHA) related to cimetidine have been reported (20–23). However, none of these cases have been tested for cimetidine-related drug-dependent antibodies. In 2010, Arndt et al. (24) reported that a 63-year-old female patient with metastatic breast cancer developed hemolytic anemia after receiving intravenous cimetidine twice. Cimetidine-dependent antibodies were detected in the serum of the patient. This was the first reported case of cimetidine-associated DIIHA that was confirmed using serological tests. However, it is unclear whether oral cimetidine can also induce the production of cimetidine-dependent antibodies and subsequently cause DIIHA.

Here, we report that a 44-year-old male patient developed severe hemolytic anemia after oral administration of cimetidine. Cimetidine-dependent antibodies were detected by incubating the patient's plasma with O-type RBCs and 1 mg/mL cimetidine or incubating the patient's plasma with cimetidine-coated RBCs at 37°C, with the highest titer reaching 4,096. Drug-independent non-specific antibodies (the highest titer reached 32) were also detected. This is the second case study to serologically diagnose DIIHA caused by cimetidine-dependent antibodies. Additionally, this is the first study to report that oral cimetidine can elicit the production of cimetidine-dependent antibodies and cause DIIHA. The findings of this study indicated that the oral administration of cimetidine can also induce the production of drug-independent non-specific antibodies, which caused positive DAT results and IHA for a prolonged period in the absence of cimetidine.

### Patient Information

**Figure 1:**
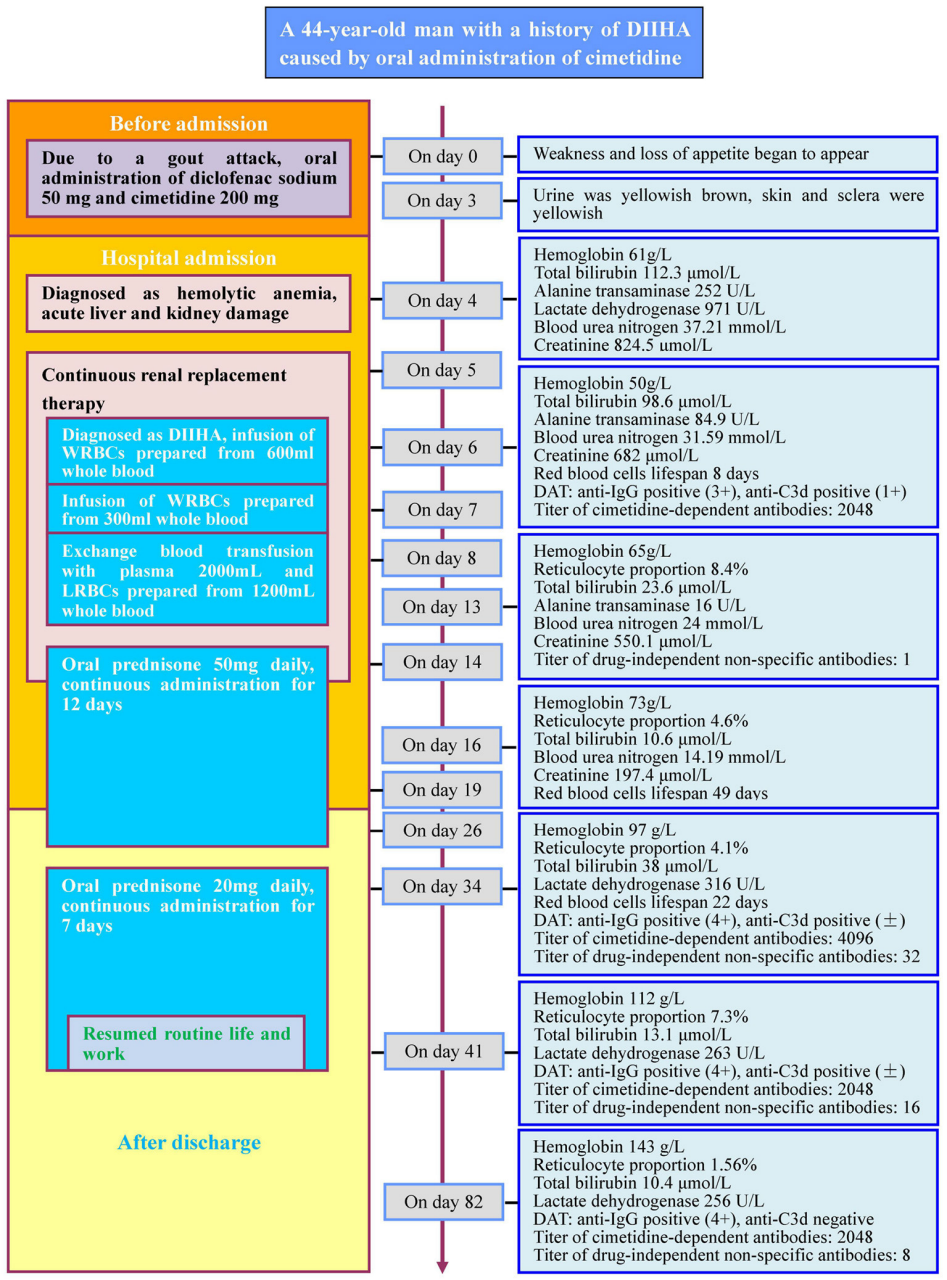
Case report timeline. DIIHA, drug-induced immune hemolytic anemia; WRBCs, washed red blood cells; LRBCs, leukocyte-reduced red blood cells. +, strong; ±, slightly strong. Red blood cells lifespan: the average survival time of red blood cells measured by using the open-breath CO breath test.

A 44-year-old male who suffered from an episode of gout (metabolic arthritis) consumed the following non-prescription drugs: diclofenac sodium sustained-release capsule (50 mg) and cimetidine capsule (200 mg). The joint pain was alleviated after medication but the patient exhibited fatigue and anorexia. Three days after drug intake, fatigue exacerbated, urine turned yellowish-brown, and the skin and sclera exhibited yellowish color. Four days after drug intake, the subject was admitted to the Dongguan Tungwah Hospital. The clinical characteristics of the patient were as follows: blood type, A/RhCcDee; glucose-6-phosphate dehydrogenase (G6PD) activity, physiological levels; expression of CD55 and CD59 on RBCs, physiological levels; thalassemia gene screening and acid hemolysis test results, no abnormalities; sucrose hemolysis test and urine hemosiderin test results, positive; bone marrow cytology, extremely active hyperplasia; paroxysmal nocturnal hemoglobinuria (PNH) clone, not detected. Color ultrasound examination revealed diffuse fatty change in the liver. The detailed results of cell and biochemical tests of blood samples collected at days 4, 6, 13, 16, 34, 41, and 82 post-drug intake are listed in Figure 1 (Case report timeline).

Five days after drug intake, the patient began to undergo continuous renal replacement therapy (CRRT). Six days after drug intake, the patient was transfused with washed RBCs (WRBCs) prepared from 600 mL of A/RhCcDee whole blood. Seven days after drug intake, the patient was transfused with WRBCs prepared from 300 mL of A/RhCcDee whole blood. Eight days after drug intake, the patient underwent exchange transfusion: 2,000 mL of A-type fresh frozen plasma was used as the replacement fluid for therapeutic plasma exchange (TPE) and the same amount of RBCs was replaced with leukocyte-reduced RBCs (LRBCs) prepared from 1,200 mL of A/RhCcDee whole blood. Fourteen days after drug intake, CRRT was stopped and prednisone (50 mg) was prescribed daily for 5 consecutive days. The patient was prescribed prednisone for 7 more days after discharge. Sixteen days after discharge (equivalent to 34 days after oral drug intake), oral prednisone (20 mg) was administered daily for seven consecutive days. Twenty-three days after discharge (equivalent to 41 days post-oral drug intake), the subject resumed routine life and work.

The patient had a history of gout for 3 years and was prescribed intermittent oral diclofenac sodium for pain relief. Additionally, the patient had no history of anemia or other chronic diseases and had not previously undergone blood transfusion. Furthermore, the patient took oral diclofenac sodium (50 mg) for pain relief due to a gout attack 10 months ago. At the same time, the patient also took one cimetidine capsule (200 mg) without any adverse reactions. The medical records of the patient did not indicate intravenous administration of cimetidine. According to the patient's statement, this was the second time cimetidine had been administered before admission.

### Assessment, Therapeutic, and Follow-Up

The patient was diagnosed with acute liver and kidney damage. Five days after drug intake, the patient underwent CRRT. During CRRT, WRBCs prepared from 900 ml of whole blood were transfused, and a blood exchange transfusion was given. Because the patient's liver and kidney functions have been significantly improved, CRRT was terminated on the 14th day after drug intake. After that, the patient returned to normal life and work after two courses of oral prednisone treatment. However, the patient's blood cells and biochemical indicators did not completely restore to normal levels until the 82nd day after drug intake.

### Timeline

The timeline of the patient's overall disease course and treatment process is shown in Figure 1.

### Definite diagnosis Based on Serological Testing

The venous blood samples were collected on days 6, 13, 34, 41, and 82 post-drug intake. Serological analysis including DAT for anti-IgG and for anti-C3d, acid elution test, irregular RBC antibody screening with saline in a tube and indirect antiglobulin test (IAT) in Coombs card were conducted on the patient's blood samples. As described by Leger et al. (25), Diclofenac sodium, cimetidine solutions, and the respective drug-coated RBCs were used to detect drug-dependent antibodies.

The DAT results for anti-IgG and anti-C3d were positive for all the venous blood samples collected on days 6, 13, 34, and 41 post-drug intake. However, on day 82 post-drug intake, the DAT results for IgG were positive while anti-C3d was negative. The results of irregular RBC antibody screening showed that IgG-type drug-independent non-specific antibodies were detected in the patient's plasma obtained from the blood samples collected on days 13, 34, 41, and 82 post-drug intake, and the acid eluent obtained from the blood samples collected on days 34, 41, and 82 post-drug intake. The detailed results of DAT and irregular RBC antibody screening are shown in Table 1.

**Table 1:** Results of DAT and irregular RBC antibodies detection in plasma and acid eluent.

**Days after post-oral drug intake**	**Direct antiglobulin test**		**Irregular red blood cell antibodies detection**		
	**anti-IgG**	**anti-C3d**	**Plasma in saline with a tube**	**Plasma IAT with coombs card**	**Acid eluent IAT with coombs card**
6 days	Positive (3+)	Positive (1+)	Negative	Negative	Negative
13 days	Positive (3+)	Positive (1+)	Negative	Drug-independent non-specific antibodies (1+)Titer = 1	Negative
34 days	Positive (4+)	Positive (±)	Negative	Drug-independent non-specific antibodies (3+)Titer = 32	Drug-independent non-specific antibodies (4+) Titer = 8
41 days	Positive (4+)	Positive (±)	Negative	Drug-independent non-specific antibodies (3+)Titer = 16	Drug-independent non-specific antibodies (4+) Titer = 16
82 days	Positive (4+)	Negative	Negative	Drug-independent non-specific antibodies (2+)Titer = 8	Drug-independent non-specific antibodies (2+) Titer = 4

*IAT, indirect antiglobulin test*.

*+, strong; ±, slightly strong*.

To test for drug-dependent antibodies we tested: (1) diclofenac sodium solution incubated with O-type WRBCs and the patient's plasma at 37°C for 1 h, (2) diclofenac sodium solution incubated with O-type WRBCs and the patient's red blood cell acid eluent at 37°C for 1 h, and (3) the patient's plasma or the acid eluent was incubated with diclofenac sodium-coated RBCs at 37°C for 1 h. No diclofenac-dependent antibodies were detected in all blood samples of the patient. However, when cimetidine solution was incubated with O-type WRBCs and the patient's plasma at 37°C for 1 h, or the patient's plasma was incubated with cimetidine-coated RBCs at 37°C for 1 h, cimetidine-dependent antibodies were detected in all blood samples of the patient. Antibodies titers detected by the two methods were the same. Further experiments indicated that the cimetidine-dependent antibodies comprised IgM and IgG. Based on the above test results, the patient was diagnosed with DIIHA caused by cimetidine. Table 2 shows the detailed results of the detection of cimetidine-dependent antibodies in samples with 1 mg/mL cimetidine solution.

**Table 2:** Test results of cimetidine-dependent antibody with RBCs and 1 mg/mL cimetidine solution (agglutination and titer).

**Days after post-oral drug intake**	**Method of observing results**	**Untreated plasma**	**2ME-plasma**	**Eluent**	**AB plasma**
6 days	Incubate for 1 h at 37°C and check with microscope	4+/titer 256	Negative	Negative	Negative
	Centrifuge in Coombs card	4+/titer 2,048	4+/titer 1,024	Negative	Negative
13 days	Incubate for 1 h at 37°C and check with microscope	4+/titer 256	Negative	NT	Negative
	Centrifuge in Coombs card	4+/titer 2,048	4+/titer 1,024	NT	Negative
34 days	Incubate for 1 h at 37°C and check with microscope	4+/titer 512	Negative	NT	Negative
	Centrifuge in Coombs card	4+/titer 4,096	4+/titer 2,048	NT	Negative
41 days	Incubate for 1 h at 37°C and check with microscope	4+/titer 128	Negative	NT	Negative
	Centrifuge in Coombs card	4+/titer 2,048	4+/titer 1,024	NT	Negative
82 days	Incubate for 1 h at 37°C and check with microscope	4+/titer 128	Negative	NT	Negative
	Centrifuge in Coombs card	4+/titer 2,048	4+/titer 1,024	NT	Negative

*2ME-plasma, 2-Mercaptoethanol-treated plasma; Eluent, Acid eluent prepared with patient's red blood cells; AB plasma, AB type healthy human plasma with negative results of irregular red blood cell antibody screening test; WRBCs, O-type washed red blood cells prepared by healthy people with negative direct antiglobulin test results. NT, no tested*.

*+, strong*.

Cimetidine powder was used to prepare aqueous solutions at multiple concentrations. Then, cimetidine solutions were incubated with O-type RBCs at 37°C or room temperature for 1 h to prepare cimetidine-coated RBCs. Additionally, O-type RBCs were incubated with cimetidine injection at multiple concentrations at 37°C or room temperature for 1 h to prepare cimetidine-coated RBCs; some RBCs hemolyzed (Table 3). All unhemolyzed cimetidine-coated RBCs were used to detect cimetidine-dependent antibodies. Table 4 shows the agglutination titers of the untreated plasma or 2-Mercaptoethanol (2ME)-treated plasma obtained from the blood samples collected on day 6 post-drug intake incubated with cimetidine-coated RBCs prepared under different conditions for 1 h at 37°C (centrifugation in a test tube or centrifugation in a Coombs card did not show hemolysis). The test results suggest that the cimetidine-coated RBCs prepared by incubating 30 or 20 mg/mL cimetidine solution with RBCs at room temperature for 1 h had the same sensitivity as that in 1 mg/mL cimetidine solution for detecting cimetidine-dependent antibodies.

**Table 3:** Appearance of cimetidine-coated RBCs prepared by mixing different dosage forms and different concentrations of cimetidine with O-type RBCs and incubating at 37°C or room temperature for 1 h.

**Concentration of drug-coated RBCs**	**Cimetidine powder**		**Cimetidine injection**	
	**Incubate at 37^**°**^C**	**Incubate at room temperature**	**Incubate at 37^**°**^C**	**Incubate at room temperature**
RBCs coated with 40 mg/mL solution	CH	PH	PH	PH
RBCs coated with 30 mg/mL solution	PH	PH	PH	NH
RBCs coated with 20 mg/mL solution	PH	PH	PH	NH
RBCs coated with 15 mg/mL solution	NH	NH	NH	NH
RBCs coated with 10 mg/mL solution	NH	NH	NH	NH
RBCs coated with 5 mg/mL solution	NH	NH	NH	NH

*CH, complete hemolysis; PH, partial hemolysis; NH, no obvious hemolysis*.

**Table 4:** Results of cimetidine-dependent antibodies titers detection with cimetidine-coated RBCs for the sample collected at 6 days after the patient oral administration of diclofenac sodium and cimetidine.

**Test**	**Untreated plasma**		**2ME-plasma**	
	**Direct centrifugal microscopy**	**Centrifuge in coombs card**	**Direct centrifugal microscopy**	**Centrifuge in coombs card**
**RBCs treated with cimetidine injection solution at 37** **°** **C**				
RBCs coated with 40mg/mL cimetidine	ND	ND	ND	ND
RBCs coated with 30 mg/mL cimetidine	ND	ND	ND	ND
RBCs coated with 20 mg/mL cimetidine	ND	ND	ND	ND
RBCs coated with 15 mg/mL cimetidine	256	2,048	Negative	1,024
RBCs coated with 10 mg/mL cimetidine	256	2,048	Negative	512
RBCs coated with 5 mg/mL cimetidine	128	1,024	Negative	512
**RBCs treated with cimetidine injection solution at room temperature**				
RBCs coated with 40 mg/mL cimetidine	ND	ND	ND	ND
RBCs coated with 30 mg/mL cimetidine	256	2,048	Negative	1,024
RBCs coated with 20 mg/mL cimetidine	256	2,048	Negative	1,024
RBCs coated with 15 mg/mL cimetidine	256	1,024	Negative	512
RBCs coated with 10 mg/mL cimetidine	256	1,024	Negative	512
RBCs coated with 5 mg/mL cimetidine	128	1,024	Negative	256

*2ME-plasma, 2-Mercaptoethanol-treated plasma; WRBCs, O-type washed red blood cells prepared by healthy people with negative direct antiglobulin test results; ND, not detectable (was not used for antibody testing because of severe hemolysis after treatment of red blood cells with drug)*.

## Discussion

The male patient had no history of anemia and blood transfusion but was diagnosed with severe hyperbilirubinemia and hemolytic anemia within 4 days of oral administration of diclofenac sodium (50 mg) and cimetidine (200 mg) along with hepatic and renal damages. Although the color ultrasound examination revealed a diffuse fatty change in the liver, the patient had no history of hepatic insufficiency or jaundice. Drugs can potentially aggravate liver damage and hyperbilirubinemia and upregulate the levels of liver enzymes. However, drug-induced liver damage does not rapidly develop into hemolytic anemia. G6PD deficiency can induce acute hemolysis by oxidizing food or drugs (26–29). A five-year retrospective study by Hagag et al. (29) demonstrated that diclofenac sodium is one of the most common drugs that induce acute hemolysis in patients with G6PD deficiency. In this study, the patient exhibited physiological levels of G6PD activity. This indicated that hemolytic anemia was not caused by G6PD deficiency. The patient also exhibited some clinical characteristics of PNH. However, cytological analysis of bone marrow revealed that the RBCs exhibited physiological expression levels of CD55 and CD59. Thus, PNH-induced hemolytic anemia was ruled out (30–32).

The results of DAT for anti-IgG and anti-C3d of the patient after oral administration of diclofenac sodium and cimetidine were all positive. These results were partially consistent with the serological characteristics of autoimmune hemolytic anemia (AIHA) (33–37) and DIIHA (10–12). As no RBC antibodies were detected in the plasma and acid eluent during the severe hemolysis period, AIHA was ruled out (33–37). Previous studies have reported diclofenac-induced (2, 38–41) or cimetidine-induced (2, 24) DIIHA. This study subject had a history of gout for 3 years and had intermittently consumed diclofenac sodium for pain relief. The patient had consumed cimetidine capsules and diclofenac sodium for gout attacks 10 months ago. This indicated that diclofenac sodium or cimetidine may be etiological agents for DIIHA.

We collected the remaining two drug capsules from the patient to prepare drug solutions and drug-coated RBCs. Drug-coated RBCs or RBCs incubated with the drug solution were used to detect the presence of diclofenac-dependent or cimetidine-dependent antibodies in the plasma. The antibody may be directed against the non-target drug component in the powder in the capsule instead of the target drug. To rule this out, a drug solution was also prepared using cimetidine solution used for injections. The cimetidine-dependent antibodies were detected in the plasma. In contrast, diclofenac-dependent antibodies were not detected in the plasma. After the cessation of cimetidine, the patient received CRRT, blood transfusion, exchange blood, and prednisone treatment. Based on alanine transaminase (normal range ≤41 U/L) and creatinine (normal range 62–106 umol/L), the hepatic and renal functions were significantly improved 13 and 16 days after cimetidine intake. The hemoglobin levels began to increase 16 days after cimetidine intake without blood transfusion. These clinical manifestations and serological findings indicated that the cimetidine-dependent antibodies produced by the patient after oral administration of cimetidine caused DIIHA. As the patient exhibited a diffuse fatty change in the liver, liver compensatory function was reduced. Additionally, hemolysis further aggravated liver damage and consequently resulted in hyperbilirubinemia and elevated liver enzymes. The patient had no history of kidney disease. Thus, renal damage may be due to hemolysis.

Several studies have reported cases of cimetidine-induced immunohemolytic anemia. However, only one patient was serologically confirmed to be a case of DIIHA caused by cimetidine-dependent antibodies (24). The ability of oral cimetidine to elicit the production of cimetidine-dependent antibodies and consequently cause DIIHA has not been previously examined. The clinical pharmacokinetic analysis of cimetidine revealed that 70% of oral cimetidine enters the blood circulation and that the chemical structure is not modified (42). This is the first study to demonstrate that oral cimetidine can produce cimetidine-dependent antibodies and cause DIIHA.

Drug-induced antibodies include drug-dependent antibodies and drug-independent antibodies (12, 25). Among more than 140 drugs that have been reported to cause DIIHA, most drug-dependent antibodies can be detected with drug-coated RBCs and/or RBCs incubated with soluble drug solutions (2, 25). Drug-independent antibodies, which are difficult to be distinguished from warm auto-antibodies, can be detected with RBC antibody screening tests without drug solutions or drug-coated RBCs (2, 25). The drug-dependent antibody test was established by Leger et al. (25) and the concentration of the drug solution used in this test is 1 mg/mL. To coat the RBCs, the RBCs are incubated with 40 mg/mL drug solution at 37°C for 1 h (except for preparing penicillin-coated RBCs). However, Arndt et al. (24) reported that the incubation of RBCs with 40 mg/mL cimetidine at 37°C for 1 h resulted in severe hemolysis and that this method does not yield a sufficient number of cimetidine-coated RBCs that can be used to detect cimetidine-dependent antibodies. Hence, cimetidine-coated RBCs were prepared by incubating RBCs with 15 mg/mL cimetidine at room temperature for 1 h. These RBCs did not undergo hemolysis and can be used to detect cimetidine-dependent antibodies.

The direct agglutination titer of the plasma (collected on day 6 post-oral cimetidine intake) and uncoated O-type RBCs in the presence of 1 mg/mL cimetidine solution was 256, while added to the Coombs card as 2,048. The direct agglutination test of the 2ME-treated plasma yielded negative results. The agglutination titer added to the Coombs card was 1,024. This indicated that the cimetidine-dependent antibodies comprised both IgM and IgG components. The cimetidine-coated RBCs prepared by incubating 30 or 20 mg/mL cimetidine solution with RBCs at room temperature for 1 h had a high sensitivity for detecting cimetidine-dependent antibodies. The Ig types and titers of cimetidine-dependent antibodies detected in the plasma were consistent with the results of examining cimetidine-dependent antibodies in the presence of cimetidine solution. Therefore, we recommend that 20–30 mg/mL cimetidine solution incubated with RBCs at room temperature is ideal for preparing cimetidine-coated RBCs for the detection of cimetidine-dependent antibodies.

This study also used Coombs card to detect drug-independent non-specific antibodies. The titers of drug-independent non-specific antibodies in plasma samples collected on days 13, 34, 41, and 82 post-drug intake were 1, 32, 16, and 8, respectively. However, drug-independent antibodies were not detected using the saline tube test. The serological properties of drug-independent non-specific antibodies were similar to those of non-specific warm auto-antibodies. We speculate that the patient who developed IHA after receiving cimetidine treatment reported by Rotoli et al. (20) could be a case of cimetidine-induced AIHA caused by drug-independent non-specific antibodies.

Currently, there are not effective therapeutic strategies for DIIHA. The discontinuation of related drugs is essential to avoid further hemolysis (43). In this study, patient with cimetidine-induced DIIHA was prescribed prednisone, CRRT, TPE, and RBC exchange treatments, which achieved immediate satisfactory effects. However, the hemoglobin levels had increased to 97 g/L when the patient returned to the clinic 16 days after discharge (34 days post-cimetidine intake) although liver enzymes and renal function had returned to physiological levels. Moreover, the titer of cimetidine-dependent antibody increased from 2,048 (before discharge) to 4,096 (after discharge). Additionally, the titer of drug-independent non-specific antibodies increased from 1 (before discharge) to 32 (after discharge). Furthermore, the results of DAT for anti-IgG were stronger after than before discharge, with an RBC lifespan of 22 days. The levels of total bilirubin and lactate dehydrogenase were higher than the reference physiological level, while the reticulocyte proportion was 4.1%. This suggested that the patient exhibited hemolytic anemia. Based on the previous understanding of DIIHA, drug-dependent antibodies can cause hemolysis only when the related drugs are in circulation. The hemolysis is alleviated when the drugs in the circulating blood are completely metabolized and all the RBCs that are bound with the drug-dependent antibody-related drug are hemolyzed. The elimination half-life of cimetidine is only 2 h (42). Hence, cimetidine must have been eliminated from the bloodstream after 34 days of cimetidine intake. Moreover, the RBCs with bound cimetidine should have been completely hemolyzed. Therefore, the occurrence of hemolysis even after 34 days of cimetidine intake should not be related to cimetidine-dependent antibodies. Instead, hemolysis may be related to the first detection of the drug-independent non-specific antibodies 13 days after cimetidine intake. The drug-independent non-specific antibodies that continue to increase after discharge from the hospital bind to RBCs and yield positive DAT results. Thus, cimetidine-independent IHA and positive DAT result can persist for a prolonged period. The titers of both cimetidine-dependent antibodies and drug-independent non-specific antibodies decreased 41 days after the cessation of cimetidine and the patient returned to routine life and work. The hemoglobin levels returned to physiological levels 82 days after the cessation of cimetidine. The duration of anti-IgG positive DAT results remains to be followed up.

Drugs administered intravenously can bind to RBCs. And in the reported cases, there is more DIIHA related to intravenous administration than DIIHA related to oral administration (1–12). However, many drugs enter the blood circulation through oral administration, the molecular structure and chemical properties of the drugs have not changed. And it has been reported that the metabolites of drugs can also cause DIIHA (25). There is currently no evidence that the risk of DIIHA from oral administration is lower than that of intravenous administration.

## Conclusions

This study reported the second case of DIIHA caused by cimetidine-dependent antibodies, which was confirmed using serological methods. Additionally, this is the first study to report that oral cimetidine can elicit the production of cimetidine-dependent antibodies and cause DIIHA. Cimetidine-dependent antibodies can be detected using RBCs incubated with cimetidine solution or cimetidine-coated RBCs. We recommended incubating O-type RBCs with 20–30 mg/mL cimetidine solution at room temperature for 1 h to prepare cimetidine-coated RBCs for the detection of cimetidine-dependent antibodies. The findings of this study indicated that the oral administration of cimetidine can also induce the production of drug-independent non-specific antibodies, which led to positive DAT results and IHA independent of cimetidine that persisted for a prolonged period. Patients with DIIHA induced by cimetidine may necessitate clinical management plans including longer monitoring and appropriate tests even after cessation of cimetidine administration.

### Patient Perspective

The patient was shocked when he was told that he had developed cimetidine-related antibodies and that the oral administration of cimetidine caused severe hemolytic anemia. The patient said he would listen to the doctor's advice that he would no longer receive cimetidine administration in any way. Doctor's explanations have relieved him from psychological burden by the long-term damage of DIIHA.

## Data Availability Statement

The original contributions presented in the study are included in the article/supplementary material, further inquiries can be directed to the corresponding author.

## Ethics Statement

The studies involving human participants were reviewed and approved by Ethics Committee of Dongguan Tungwah Hospital. The patients/participants provided their written informed consent to participate in this study. The patient signed the informed consent and agreed to publish the findings about his case.

## Author Contributions

YJW, YW, and YLJ contributed to the conception and design of the study, data analysis, and manuscript draft preparation. YJW, YHL, and JJL prepared the draft and final manuscripts. DSW monitored the patient and conducted the treatment. YJW, YW, YLJ, GPG, and BCC performed the immunohematological test. All authors contributed to the study design, result interpretation, and preparation of the manuscript.

## Funding

This study was supported by two Key Project of Social and Scientific Development of Dongguan in 2019 (201950715046181 and 201950715007214).

## Conflict of Interest

The authors declare that the research was conducted in the absence of any commercial or financial relationships that could be construed as a potential conflict of interest.

## Publisher's Note

All claims expressed in this article are solely those of the authors and do not necessarily represent those of their affiliated organizations, or those of the publisher, the editors and the reviewers. Any product that may be evaluated in this article, or claim that may be made by its manufacturer, is not guaranteed or endorsed by the publisher.

## Abbreviations

IHA: immune hemolytic anemia
DIIHA: drug-induced immune haemolytic anaemia
AIHA: autoimmune hemolytic anemia
DAT: direct antiglobulin test
NIPA: non-immunologic protein adsorption
RBC: red blood cell
RBCs: red blood cells
WRBCs: washed red blood cells
LRBCs: leukocyte-reduced red blood cells
G6PD: glucose-6-phosphate dehydrogenase
PNH: paroxysmal nocturnal hemoglobinuria
CRRT: continuous renal replacement therapy
TPE: therapeutic plasma exchange
2ME: 2-Mercaptoethanol.
